# Adhesive and Flame-Retardant Properties of Starch/Ca^2+^ Gels with Different Amylose Contents

**DOI:** 10.3390/molecules28114543

**Published:** 2023-06-04

**Authors:** Peng Liu, Jiandi Ling, Taoyan Mao, Feng Liu, Wenzhi Zhou, Guojie Zhang, Fengwei Xie

**Affiliations:** 1 School of Chemistry and Chemical Engineering, Guangzhou University, Guangzhou Higher Education Mega Center, Guangzhou 510006, China; liu_peng@gzhu.edu.cn (P.L.); taoyanmao@gzhu.edu.cn (T.M.); 2 School of Engineering, Newcastle University, Newcastle upon Tyne NE1 7RU, UK; 3 Jiangsu Sanshu Biotechnology Co., Ltd., Nantong 226006, China

**Keywords:** starch gel, adhesive, flame retardancy

## Abstract

Starch, being renewable and biodegradable, is a viable resource for developing sustainable and environmentally friendly materials. The potential of starch/Ca^2+^ gels based on waxy corn starch (WCS), normal corn starch (NCS), and two high-amylose corn starches, G50 (55% amylose content) and G70 (68% amylose content) as flame-retardant adhesives has been explored. Being stored at 57% relative humidity (RH) for up to 30 days, the G50/Ca^2+^ and G70/Ca^2+^ gels were stable without water absorption or retrogradation. The starch gels with increasing amylose content displayed increased cohesion, as reflected by significantly higher tensile strength and fracture energy. All the four starch-based gels showed good adhesive properties on corrugated paper. For wooden boards, because of the slow diffusion of the gels, the adhesive abilities are weak initially but improve with storage extension. After storage, the adhesive abilities of the starch-based gels are essentially unchanged except for G70/Ca^2+^, which peels from a wood surface. Moreover, all the starch/Ca^2+^ gels exhibited excellent flame retardancy with limiting oxygen index (LOI) values all around 60. A facile method for the preparation of starch-based flame-retardant adhesives simply by gelating starch with a CaCl_2_ solution, which can be used in paper or wood products, has been demonstrated.

## 1. Introduction

Starch is a renewable biomaterial that is synthesized in the amyloplasts of plant cells and stored in plant organs such as tubers, roots, and seeds [1]. It is often used as a sustainable and environmentally friendly material in the food, textile, paper, packaging, and chemical industries [2,3,4]. Starch-based adhesives are one of the least expensive types of non-toxic, renewable adhesives on the market and have been used for a long time [5]. They have been historically used as a binder by ancient Egyptians for making papyrus [6].

Starch adhesives are mainly applied to wood and paper [7] and in the textile industries as binders and sizing agents [8]. However, starch adhesives usually contain other additives, such as tetraborate (borax), to increase adhesion, cohesion, and viscosity stability [9]. Chemical modification, which replaces the original hydrophilic hydroxyl groups with other groups (e.g., oxidized starch) or increases the cohesive forces between starch molecules (e.g., graft-copolymerized starch), can improve adhesive properties [10].

There are two macromolecules in starch granules, amylose and amylopectin. In native normal and waxy starches, amylopectin plays a vital role in determining properties. In recent years, because of the strong cohesive force imparted by inter-amylose chain interactions, high-amylose starch has attracted wide attention [11,12]. Based on its unique properties, such as good mechanical strength and water retention, high-amylose starch has been used to develop environmentally friendly, biodegradable materials [13], strain-sensitive batteries, and self-powered sensors [14]. However, the adhesive properties of high-amylose starch have not been previously reported, likely because it is hard to obtain homogeneous gels based on high-amylose starch. Compared with waxy and normal starches, the gelatinization of high-amylose starch is much more difficult and usually requires a high temperature and more energy to disrupt the compact granular structure and the entanglements between amylose chains [15,16,17].

The preparation of flame retardants based on starch has been explored. Although starch powder is flammable and starch gel displays poor flame retardancy [18], starch has an abundance of hydroxyl groups with high reactivity, and thus flame-retardant groups can easily be grafted onto the polymeric structure. For example, starch can be esterified using a large amount of phosphoric acid without formaldehyde to attach multiple phosphoric acid groups and then converted to ammonium salts to realize flame retardancy [19]. Further, starch [18], cellulose [20], and alginate fibers [21] modified by the presence of metal ions also exhibit good flame retardancy.

Our previous research found that a CaCl_2_ solution can totally disorganize high-amylose starch granules, leading to a homogeneous gel [22,23]. Herein, the cohesion and adhesion of starch/Ca^2+^ gels with different amylose/amylopectin ratios have been compared, and the adhesive and flame-retardant properties have been evaluated to show application potential.

## 2. Results and Discussion

### 2.1. Molecular Information of Starches

**Figure 1:**
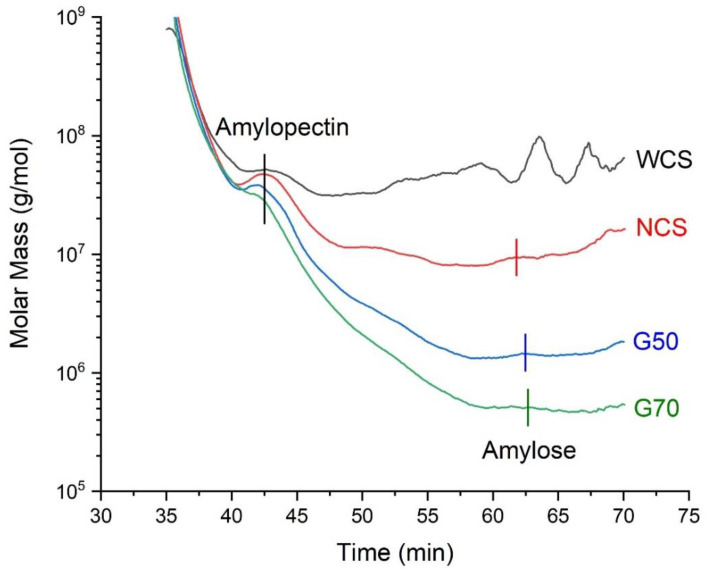
Gel permeation chromatography (GPC) profiles of the different starches (waxy corn starch (WCS), normal corn starches (NCS), and two high-amylose starches (G50 and G70)).

Figure 1 shows the gas permeation chromatography (GPC) profiles of the four native starches, with the related information tabulated in Table 1. The overall molecular mass (MM) of WCS is above 5 × 10^7^ g/mol with a peak at about 1 × 10^8^ g/mol, corresponding to the amylopectin MM of about 5 × 10^7^–1 × 10^8^ g/mol, which is consistent with previous results [24,25]. For NCS, its amylopectin MM is about 5–7 × 10^7^ g/mol, which is slightly lower than that of WCS, and its amylose MM is about 9 × 10^6^ g/mol. For the high-amylose starches G50 and G70, their amylopectin MM is about 5–6 × 10^7^ g/mol and 3–4 × 10^7^ g/mol, and the amylose MM is 2.0 × 10^6^ g/mol and 7.2 × 10^5^ g/mol, respectively. In other words, with the higher amylose content, the MM becomes lower. This trend is in agreement with previous data [26].

### 2.2. Mass and Crystalline Structure Changes of Starch/Ca^2+^ Gels

**Figure 2:**
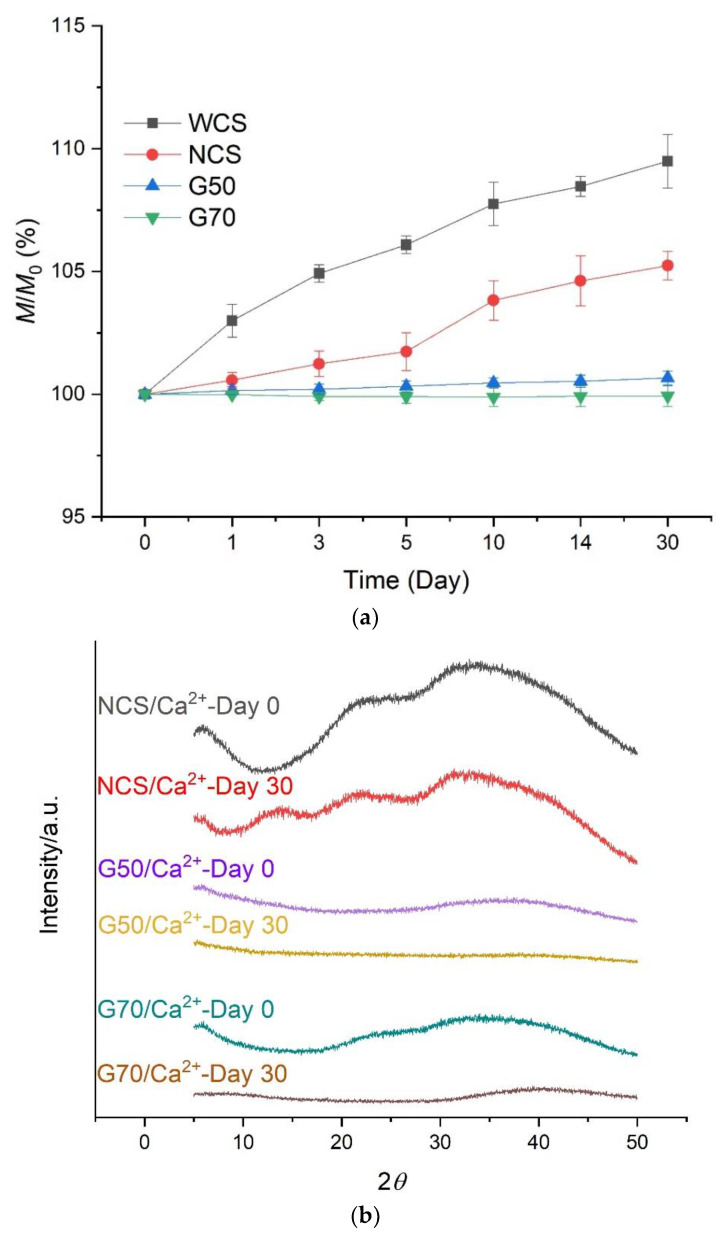
(**a**) Mass changes and (**b**) X-ray diffraction (XRD) curves of starch/Ca^2+^ gels made from different starches (waxy corn starch (WCS), normal corn starches (NCS), and two high-amylose starches (G50 and G70)) stored at 57% RH environment for up to 30 days.

In Figure 2a, *M*_0_ is the original mass of the starch gels before storage, and *M* is the real-time mass. The masses of the G50 and G70 gels almost remain unchanged during storage. The masses of the WCS/Ca^2+^ and NCS/Ca^2+^ gels increase slightly, and that of the WCS/Ca^2+^ gel is the highest among the four samples. These results show that the WCS/Ca^2+^ starch gel absorbs water easily, but when amylose content rises, the water absorption capability is weakened. Regarding this, as it is easier for linear amylose chains to entangle and interact with each other than branched amylopectin molecules, a higher amylose content can contribute to a more compact network. On the other hand, the starch/Ca^2+^ gels stored at 84% RH for 30 days (Figure S1) show increased masses, which should be contributed by the absorption of moisture by Ca^2+^. Despite this, the mass changes also prove that the starch/Ca^2+^ gel has reduced water absorption ability with increasing amylose content.

Figure 2b shows the XRD curves of the NCS, G50, and G70 starch/Ca^2+^ gels, all in a solid-like state at room temperature. Since the WCS gel is still in a fluid state (see Section 3.3), its XRD curves could not be detected. Initially, at Day 0, the crystalline peaks vanish in all XRD curves, which means that all starch granules are totally disorganized by the CaCl_2_ solution, and the gels are all fully amorphous. This is consistent with our previous results [23]. Secondly, after 30 days of storage, the curves still have no crystalline peaks, so no recrystallization (retrogradation) happens. A previous paper [27] suggested that easier recrystallization is an important trait of high-amylose starch-based materials, as linear amylose chains tend to aggregate. However, from Figure 2b, we can see that the aggregations and retrogradations between amylose chains are hindered. Regarding this, the network of gels is stable due to the coordination between starch chains and Ca^2+^.

### 2.3. Rheological Properties of Starch/Ca^2+^ Gels

**Figure 3:**
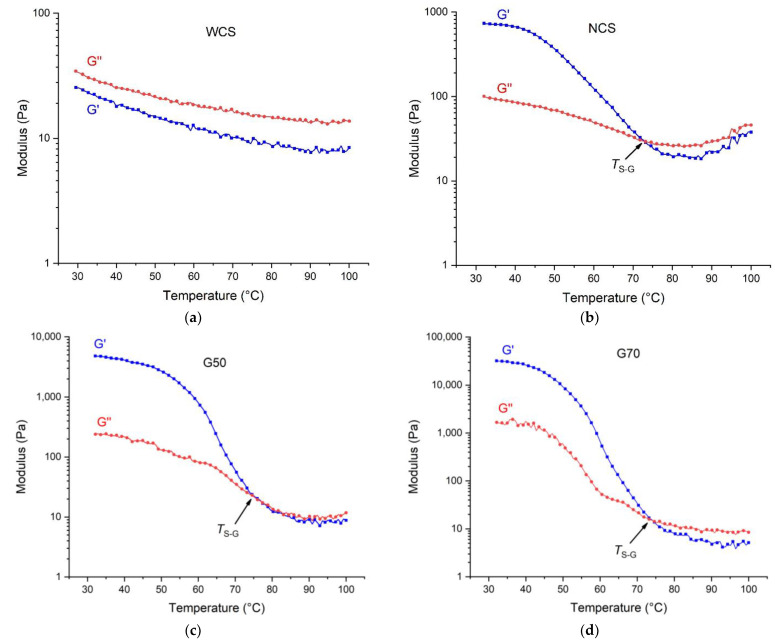
Dynamic viscoelastic properties (storage modulus (*G*′) and loss modulus (*G*″)) of the starch/Ca^2+^ gels made from different starches (waxy corn starch (WCS) (**a**), normal corn starches (NCS) (**b**), and two high-amylose starches (G50 and G70) (**c**,**d**)) under the temperature–sweep mode.

Figure 3a shows that both the storage modulus (*G′*) and the loss modulus (*G″*) of the WCS/Ca^2+^ gel are extremely low, and *G″* is higher than *G′*, indicating a more fluid-like characteristic of the amylopectin/Ca^2+^ gel at room or higher temperatures. In contrast, for the other starch/Ca^2+^ gels (Figure 3b–d), *G′* is significantly higher than *G″* at room temperature, implying an elastic and solid-like characteristic. The G70/Ca^2+^ gel, with the highest amylose content, shows the highest moduli *G′* and *G″*, and its *G′* is nearly 40 times that of the NCS/Ca^2+^ gel. In other words, with a greater amount of amylose chains, the gel shows higher elasticity.

The results indicate that the cohesive force in amylose/Ca^2+^ composites is stronger than that in amylopectin/Ca^2+^ because it is the amylose–Ca^2+^ interaction that contributes to the network with solid-like and elastic properties in starch-based gels. Specifically, Ca^2+^ can coordinate with starch through the C6 hydroxyl group in glucosides [28], and one Ca^2+^ cation can connect with multiple hydroxyl groups [29] and thus functions as a crosslinker and reinforces the intermolecular force between starch chains. For amylose, which is in the long spiral chain conformation [24], coordination can largely occur between different molecular chains, leading to intermolecular crosslinking. Thus, the presence of Ca^2+^ cations can enhance the intermolecular force in the gels based on NCS, G50, and G70. On the other hand, for amylopectin, due to its short branched chains and gel–ball conformation [30], the coordination mainly occurs on the short branched chains within the molecule, and the coordination between different macromolecules is less significant.

On the other hand, for the NCS-, G50-, and G70-based gels, with an increasing temperature, *G′* drops sharply, and *G″* decreases slightly, leading to a crossing point at a certain temperature, which is the sol–gel transition temperature (*T*_S-G_) [31]. This suggests that the elasticity of the starch/Ca^2+^ gels is eliminated upon heating, and the solid-like gel changes to a fluid-like state. In other words, the starch/Ca^2+^ gels based on NCS, G50, and G70 have a reversible temperature-dependent sol–gel transition. This transition has scarcely been reported for starch-based gels, while a similar transition of pectin or agar gels has frequently been observed [32]. Amylose is proposed to have a key role in determining the sol–gel transition of starch [33]. For the amylose/Ca^2+^ network, since the coordination ability of Ca^2+^ is not very strong [34], heating increases the kinetic energy of amylose chains and breaks the coordination bonds and the network, and thus the solid-like gel transforms into a fluid-like gel. Upon cooling, coordination and a crosslinked network form again, and so does a solid-like gel. Moreover, all the NCS/Ca^2+^, G50/Ca^2+^, and G70/Ca^2+^ gels display a *T*_S-G_ value slightly higher than 70 °C, and there are no significant differences among them with amylose content. This means that, with enough amylose content, the amylose–Ca^2+^ interaction leads to a more elastic gel, but their coordination effect has no relationship with amylose content—only with temperature, and so does the sol–gel transition.

In addition, the steady–shear rheological properties of the different starch/Ca^2+^ colloidal solutions are also detected, with the results presented in Figure S2. All the starch colloidal solutions exhibit shear-thinning behavior, and the WCS/Ca^2+^ and NCS starch/Ca^2+^ colloidal solutions show a higher viscosity than G50 and G70 ones. Regarding this, at a low concentration, the viscosity of the colloidal solution is contributed more by the hydration between starch chains and water molecules but less by the entanglement of starch chains. Since WCS and NCS have more amylopectin chains than G50 and G70, and the hydration capability of amylopectin is much stronger than that of amylose, it is reasonable to observe higher viscosity for the WCS and NCS colloidal solutions.

### 2.4. Tensile Properties of Starch/Ca^2+^ Gels

Cohesive force, which means the interactions of the same molecules within a bulk gel, plays an essential role in sticking the gel together to make it whole [35]. Here, tensile properties were used to reflect cohesive force.

**Figure 4:**
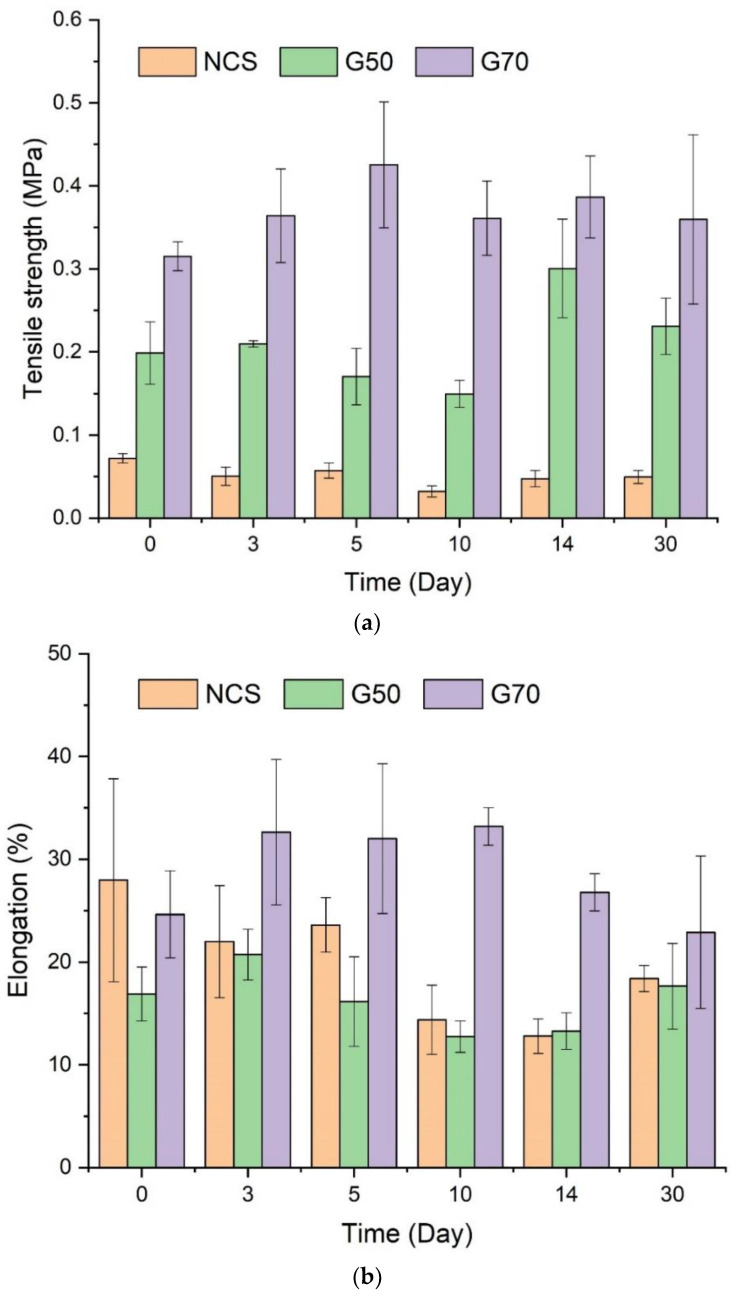

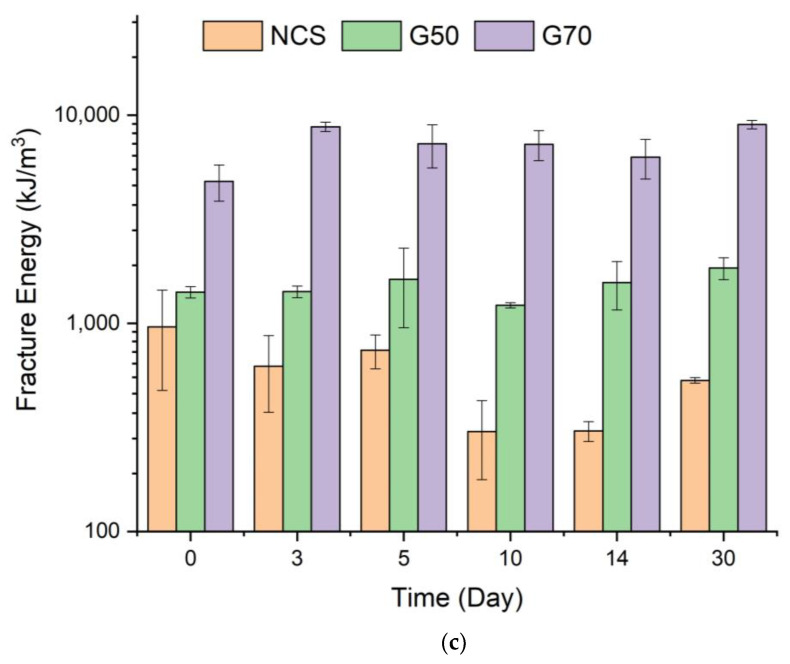
Tensile mechanical properties of the starch/Ca^2+^ gels made from different starches (waxy corn starch (WCS), normal corn starches (NCS), and two high-amylose starches (G50 and G70)) stored at 57% relative humidity (RH) up to 30 days: (**a**) tensile strength, (**b**) elongation at break, and (**c**) fracture energy.

Figure 4 shows the tensile strength (a), elongation at break (b), and fracture energy (c) of the NCS, G50, and G70 starch/Ca^2+^ gels. The tensile properties of the WCS/Ca^2+^ gel cannot be detected because it is in a fluid-like state at room temperature. The G70/Ca^2+^ gel has the highest tensile strength, elongation at break, and fracture energy, suggesting it has the densest network and the strongest cohesive force. Following it, the G50/Ca^2+^ gel has higher tensile strength and fracture energy than those of NCS/Ca^2+^ gel, which can also be attributed to the strong network and cohesive force formed by the amylose/Ca^2+^ composite. A similar trend of mechanical properties is shown in a previous study on starch-based films with different amylose contents prepared by extrusion [36]. Moreover, the fracture energies of the G70/Ca^2+^ and G50/Ca^2+^ gels are almost unchanged throughout the whole test duration. Nevertheless, the fracture energy of the NCS/Ca^2+^ gel decreases gradually with time. This should be contributed by water absorption during storage. Specifically, from Figure 2a, the NCS/Ca^2+^ gel can absorb water gradually. Additionally, since water molecules have high polarity, they can act as a plasticizer or displacer to destroy the hydrogen bonds between starch chains, so the fracture energy drops.

From the results of tensile properties, it can be deduced that it is the amylose/Ca^2+^ interaction that contributes to the cohesive force of starch-based gels, and with higher amounts of amylose/Ca^2+^ in the gel, the cohesive force is stronger.

### 2.5. Adhesive Ability of Starch/Ca^2+^ Gels

**Figure 5:**
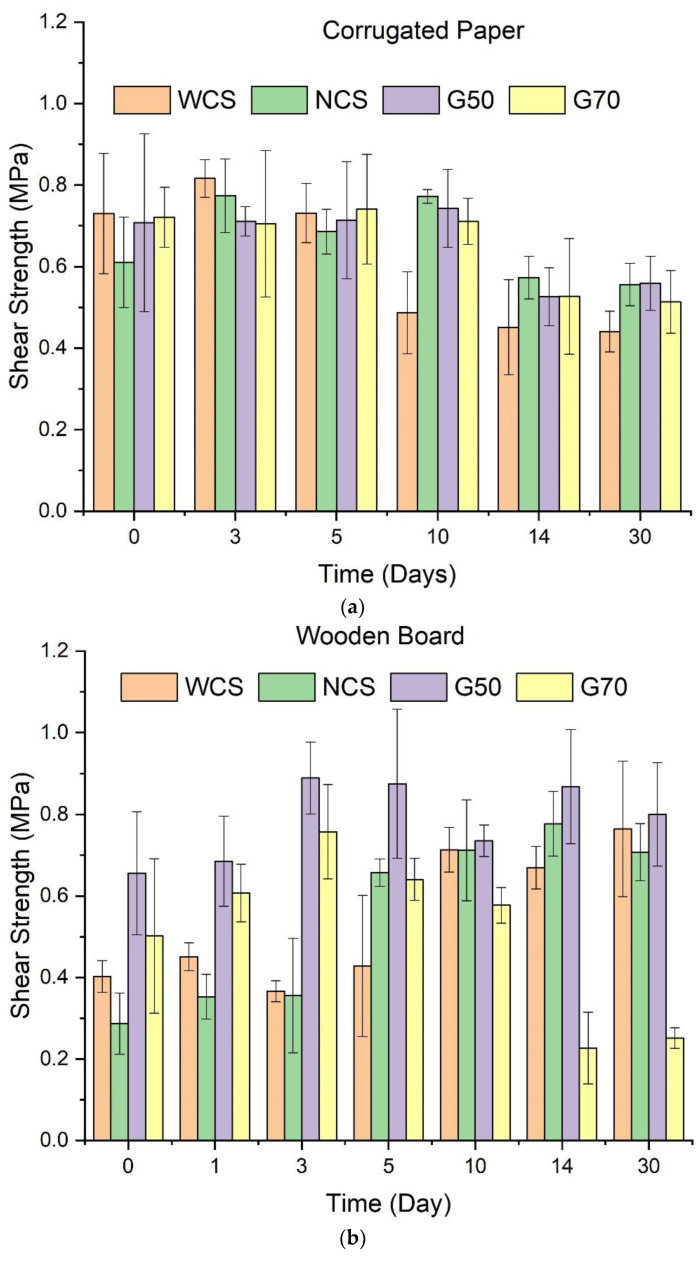
Shear strength of the starch/Ca^2+^ gels made from different starches (waxy corn starch (WCS), normal corn starches (NCS), and two high-amylose starches (G50 and G70)) on corrugated paper (**a**) and wooden board (**b**) stored at 57% relative humidity (RH) for up to 30 days.

The adhesive abilities of starch/Ca^2+^ gels with different amylose contents are reflected by the shear strength of the corrugated paper (Figure 5a) and wooden board (Figure 5b) adhered by the gels and then stored under 57% RH for 30 days.

At room temperature, the starch gels are in a solid state and have a very weak adhesive property. They can be used as adhesives only when heated and transformed to a fluid state, so they can be coated on paper or wood boards easily and homogeneously. Regarding this, the adhesive property of starch/Ca^2+^ gels mainly comes from the hydroxyl groups on the gel surface. When they change to a solid state, most hydroxyl groups on the material surface will be embedded into the inside of the gel due to the hydrophilicity and hydrophobicity in the gas–liquid interface. Only when they are in a fluid state can the hydroxyl groups be exposed to form hydrogen bonds with the cellulose and hemicellulose on the surface of paper or wood.

Figure 5a shows that, during the first 5 days, there are no obvious differences in adhesive force (reflected by shear strength) of the starch gels (all around 0.7 MPa). The torn sections of the bonded corrugated paper are covered by paper residues, not the starch gel (Figure S3). This shows that the tearing comes from the weak original adhered parts within the corrugated fiberboard, not the starch gels sticking the paper pieces, meaning that the adhesive ability of the starch/Ca^2+^ gels on corrugated paper is stronger than the paper. In addition, the shear strength of the original adhesive force of corrugated paper is 0.74 ± 0.15 MPa, which is consistent with the results.

On the other hand, after 10 days of storage, the shear strength of the WCS/Ca^2+^ starch gel reduces obviously, but the shear strength of the other gels is unchanged. This should be attributed to the high-water absorption ability of WCS/Ca^2+^ gel, which is consistent with the results in Figure 2a because a high moisture content weakens the adhesive force of the gel by acting as a plasticizer or displacer between the paper surface and the starch/Ca^2+^ gel. Moreover, after a long storage period (Day 14), all the shear strengths of the NCS/Ca^2+^, G50/Ca^2+^, and G70 starch/Ca^2+^ gels dropped, to be less than 0.6 MPa. Figure 2a shows that during storage, there are no significant changes in water absorption for the starch/Ca^2+^ gels, so their adhesive ability should not change significantly. Considering this, the reduction should be attributed to the decreased cohesion within the corrugated fiberboard. In other words, the corrugated paper would absorb water after a long time at 57% relative humidity (RH), so its inner cohesion decreases.

In Figure 5b, on the surface of the torn section, only the residues of the starch/Ca^2+^ gels were observed, and the surface of the bonded wood was not torn apart (Figure S4). Therefore, these shear strength values reflect the adhesive abilities of the gels. Comparing Figure 5a with Figure 5b, the shear strength of the corrugated paper is higher than that of the wooden board on Day 0. However, after storage, the shear strength of wood increases gradually and finally surpasses that of paper (except for the G70/Ca^2+^ gel). This can be attributed to the slow formation of hydrogen bonds between the starch gels and wood. Different from paper, which is mainly composed of cellulose, wood is composed of micro-fibrils of cellulose (40–50%) and hemicellulose (15–25%) impregnated with lignin (15–30%) [37]. Lignin confers hydrophobicity and hinders the formation of hydrogen bonds between the starch gels and wood. As a result, hydrogen bonds are formed slowly, and the adhesive force is strengthened gradually.

Secondly, within the short storage time, the G50/Ca^2+^ gel has the strongest adhesive ability on wooden boards. It is reasonable that the shear strength of the G50/Ca^2+^ gel is higher than that of the WCS/Ca^2+^ and NCS/Ca^2+^ gel because more amylose contributes to higher cohesion. On the other side, the reduction in shear strength of the G70/Ca^2+^ gel illustrates that if the amylose content is even higher (70%), most starch chains will be used to improve cohesion, so the hydrogen bonding on the interface between the gel and wooden boards, which contributes to the adhesive force directly, is weakened. In addition, from the images of the torn section surfaces (Figure S4), we can see that the residues are well distributed for the WCS/Ca^2+^, NCS/Ca^2+^, and G50/Ca^2+^ gels, but for the G70/Ca^2+^ gel, the residues are large blocks, which proves the easy strip and the weak adhesive force. Moreover, with extended storage, the adhesive ability of G70/Ca^2+^ gel falls sharply and is far weaker than the other gels.

Moreover, after prolonged storage (10 days), the differences in adhesive ability among the WCS/Ca^2+^, NCS/Ca^2+^, and G50/Ca^2+^ starch gels on wooden boards are narrowed. This shows that when the hydrogen bonds reach the maximum, the adhesive ability of the starch-based gels has no apparent relationship with amylose content, although more amylose contributes to stronger cohesion. Regarding this, a possible reason could be that the amount of the starch gels coated onto wood is small, which only forms a thin layer on the surface of wooden boards (about 40–100 μm as shown by SEM photos, Figure S5); therefore, the cohesion of the gels has little influence on their adhesive ability.

All in all, the adhesive strength of starch/Ca^2+^ gels can meet the requirements for paper and wood. Specifically, when used as paper adhesives, their adhesive forces are stronger than the cohesion within the paper. When used as wood adhesives, their shear strengths are higher than 0.8 MPa (except G70 gel). The Chinese National Standards GB 9648-2015 state that the minimum adhesive strength of the wood adhesive is 0.7 MPa. Therefore, the starch/Ca^2+^ gels can meet the requirements for use.

Besides paper and wood, the starch/Ca^2+^ gels were also tested to bond glass, PE film, and iron sheets. However, the gels do not have the adhesive ability on these materials, likely due to the lack of polar groups on the surface of these materials to bind with the gels.

### 2.6. Flame-Retardant Properties of Starch/Ca^2+^ Gels

Previous researchers [21] have pointed out that the introduction of metal ions (e.g., Na^+^, K^+^, and Li^+^) into carboxymethyl starch can improve flame retardancy. Here, we considered that the high concentration of Ca^2+^ in the starch/Ca^2+^ gels may also impart them with flame retardancy.

The limiting oxygen index (LOI) of the starch/Ca^2+^ gels is around 60. In comparison, the LOI of native NCS starch powder is only 20. It was proposed previously that with a LOI value less than 21, the sample ignites easily, burns rapidly, and is considered a flammable material; when the LOI value is between 24 and 27, the sample is difficult to ignite, and is considered a combustible material; and when the LOI value is above 27, the sample is considered as a non-flammable material [38]. Generally, a higher LOI value indicates that the material is more difficult to combust, and all the starch/Ca^2+^ gels can be considered non-flammable.

Video Clip S1 shows the flame-retardant application of the G50/Ca^2+^ gel in paper. Specifically, the gel adhered to the middle zone of the two pieces of paper. Outside the coating zone, two pieces of paper were not pasted together. When the paper was ignited, the flame was fierce. However, when the flame burned to the coating zone, the carbonization zone appeared quickly to prevent the further spreading of the flame. The flame could not pass through the carbonization zone, and when all the paper in the uncoated area was burned out, the flame gradually extinguished.

Video Clip S2 shows the flame-retardant application of the G50/Ca^2+^ gel in wood. The gel was coated on the surface of the wood, which was compared with a control sample without coating. Both samples are stored under 57% RH for 1 week. After that, the flame-spraying gun was used to light the wood. When the flame contacted the wood coated with the gel, the contact point carbonized rapidly to prevent the flame from spreading. After removing the flame-spraying gun, the flame extinguished rapidly without any residue. However, the flame on the control sample spread quickly, and when the flame-spraying gun was removed, the flame did not extinguish but continued to spread.

Based on previous studies about the flame retardancy of Na^+^- and K^+^-grafted starch [18] and Ca^2+^-grafted cellulose [20], it is deduced that during heating, Ca^2+^ is easy to react with oxygen to produce oxides. This process consumes oxygen, leads to the insufficient combustion of starch adhesives, and impels them to form incombustible carbon film. The carbon film blocks the contact between combustible materials (paper and wood) and air, so the flame gradually extinguishes.

## 3. Materials and Methods

### 3.1. Materials

Waxy corn starch (WCS) and normal corn starch (NCS) were purchased from Dezhou Dacheng Food Co., Ltd. (Dezhou, China). Two kinds of high-amylose corn starch, labeled G50 and G70, were supplied by Gansu Kunlun Biochemical Co., Ltd. (Zhangye, China) and Hainan Shanliang Technology Co., Ltd. (Haikou, China), respectively. The information on amylose content is listed in Table 1.

All chemical reagents, including anhydrous calcium chloride, sodium bromide, and potassium chloride, were purchased from Shanghai Macklin Biochemical Technology Co., Ltd. (Shanghai, Country) and were analytically pure. Lithium bromide, silicone oil (DC 200), and an amylose standard were purchased from Sigma-Aldrich (Shanghai, China) Trading Co., Ltd. (Shanghai, Country); dimethyl sulfoxide was purchased from ANPEL Laboratory Technologies Inc. (Shanghai, China).

### 3.2. Molecular Information of Starches

Native starch (5 mg) was thoroughly mixed with 5 mL of a dimethyl sulfoxide (DMSO) solution containing lithium bromide (0.5% *w*/*w*) (LiBr/DMSO) and heated at 80 °C using a stirrer heater for 3 h. After that, the molecular mass of various fractions was measured using SEC-MALLS-RI (U3000, Thermo, Waltham, MA, USA). The weight- and number-average molecular masses (*M*_w_ and *M*_n_) and polydispersity index (*M*_w_/*M*_n_) of various fractions in the LiBr/DMSO (0.5% *w*/*w*) solution were measured on a DAWN HELEOS-II laser photometer (He-Ne laser, λ = 663.7 nm, Wyatt Technology Co., Santa Barbara, CA, USA) equipped with three tandem columns (300 × 8 mm, Shodex OH-pak SB-805, 804, and 803, Showa Denko K. K., Tokyo, Japan), which were held at 60 °C using a model column heater. The flow rate was 0.3 mL/min. A differential refractive index detector (Optilab T-rEX, Wyatt Technology Co., Santa Barbara, CA, USA) was simultaneously connected to give the concentration of fractions and the *d*_n_/*d*_c_ value. The *d*_n_/*d*_c_ value of the fractions in the DMSO solution was determined to be 0.07 mL/g. Data were acquired and processed using ASTRA6.1 (Wyatt Technology).

### 3.3. Amylose Contents in Starch Granules

The amylose content of the starch was determined by the iodine–starch colorimetry method. Specifically, the iodine/potassium iodide solution was prepared by dissolving 2.0 g of KI and 0.2 g of I_2_ into 100 mL of distilled water. After that, amylose solutions were prepared by adding 0.100 g of the standard amylose or the starch sample into 10 mL of a KOH solution (0.1 mol/L), followed by heating at 80 °C for 30 min. Subsequently, 0.3 mL of the amylose or starch sample solution was mixed with 0.5 mL of the iodine/potassium iodide solution, and the volume was adjusted to 50 mL by adding distilled water. At last, the absorbance was detected by a spectrophotometer (UV2600, Shimadzu, Kyoto, Japan).

### 3.4. Preparation of Starch/Ca^2+^ Gels

First, 50 g of anhydrous CaCl_2_ was added to 100 mL of deionized water to obtain a 33% (*w*/*w*) CaCl_2_ solution. Then, 40 g of the starch powders was added into the CaCl_2_ solution and stirred at 85 °C and 60 rpm using a top–down mechanical mixer for 40 min to obtain a homogeneous starch/CaCl_2_ solution. During the heating, the reaction system is sealed off by a cover to prevent water evaporation. When the solutions were cooled down to room temperature, starch/Ca^2+^ gels were obtained. The moisture content of the starch/Ca^2+^ gels was measured to be about 50% by heating them at 110 °C until a constant weight.

### 3.5. Mass and Crystalline Structure Changes of Starch/Ca^2+^ Gels during Storage

#### 3.5.1. Water Retention Property

The starch/Ca^2+^ gels prepared were first stored at 57% relative humidity (RH) for 24 h to eliminate the influence of sample preparation. After that, they were stored under 57% RH or 84% RH (achieved by saturated NaBr and KCl salt solutions, respectively), and their masses were recorded daily.

#### 3.5.2. X-ray Diffraction (XRD)

The crystallinity of starch/Ca^2+^ gels was analyzed using an Xpert PRO diffractometer (PANalytical B.V., Almelo, The Netherlands) operated at 40 mA and 40 kV with Cu Kα radiation (wavelength, 0.1542 nm). The scanning was performed from 5 to 50° 2*θ* at a speed of 10°/min and a step size of 0.033°.

### 3.6. Rheological Properties of Starch/Ca^2+^ Gels

#### 3.6.1. Oscillatory Frequency–Sweep Test

An MCR 92 rheometer (Anton Paar GmbH, Graz, Australia) with a 50 mm parallel plate and a temperature control system was used to detect the dynamic viscoelastic behavior of the starch/Ca^2+^ gels. The distance between the two plates was set to 1.5 mm, the strain was 0.1%, the frequency was 1 Hz, and the temperature ramp was from 30 to 100 °C. Silicone oil (DC 200) was placed around the edge of the measuring cell to maintain the moisture content of the sample.

#### 3.6.2. Steady–Shear Test

By reducing the starch concentration in the starch/Ca^2+^ gels (10 g of the starch was added into the 150 g 33% CaCl_2_ solution), colloidal solutions were obtained. The MCR 92 rheometer was used to detect their shear-thinning behavior. The distance between the two plates was set to 1 mm, and the shear rate was set from 0.01 to 100 s^−1^ at 25 °C. The power–law model was used to fit the shear-thinning behavior.

### 3.7. Tensile Properties of Starch/Ca^2+^ Gels

A texture analyzer with a 50 kg load cell was used to measure the tensile properties of the starch/Ca^2+^ gels at room temperature. The samples were cut into a dumbbell shape of 35 × 2 mm, following the Chinese National Standard GB/T 528-2009. The extension rate was kept at 0.5 mm/s. The tensile strength and elongation at the break were evaluated by the instrument software. The fracture energy, the area enclosed by the stress–strain curves, was calculated by Origin 2018.

Before detection, all the gels were moisture-equilibrated at 57% RH for 24 h, which was recorded as Day 0. After that, the tensile properties of the gels were detected at certain storage time points. At least five tests (based on five specimens) were performed for each sample.

### 3.8. Adhesive Properties of Starch/Ca^2+^ Gels

The shear adhesive strength of the starch/Ca^2+^ gels was tested according to the Chinese national standard (GB/T 33333-2016). Specifically, all gels were kept at 85 °C and in a solution state. The pieces of corrugated paper or wooden board (*Paulownia fortunei*) with a size of 4 cm × 2.5 cm were selected as the bonded samples. Specifically, 0.05 g of the gel was coated evenly on the sample surface with a coating area of 2.5 cm^2^ (1 cm × 2.5 cm), and then another sample was bonded on it. After that, the samples were stressed by a 1 kg weight for 15 min, then the weight was removed, and the samples were dried at 75 °C for 45 min. Subsequently, they were placed at 57% RH for 24 h, which was recorded as Day 0. After that, the adhesive properties (shear strength) of the gels on the bonded corrugated paper or wooden board were analyzed at certain storage time points. For each sample, at least five tests (based on five specimens) were performed.

The texture analyzer with a 50 kg load cell was used to evaluate the adhesive ability. The two ends of the bonded corrugated paper or wooden board were fastened by the fixtures of the texture analyzer. The samples are tightened by the clamp with a grooved surface to prevent the samples from slipping. The extension rate was 2 mm/s. After detection, the shear strength was detected.

### 3.9. Morphology of the Section of Bonded Wooden Boards

The sections of bonded wooden boards were imaged by a scanning electron microscope (Zeiss Sigma 300, Carl Zeiss AG, Oberkochen, Germany) with an accelerating voltage of 10 kV.

### 3.10. Limiting Oxygen Index of Starch/Ca^2+^ Gels

LOI is the minimum concentration of oxygen in an oxygen/nitrogen gas stream mixture to maintain combustion, which was used to assess the ignition and ease of extinction of a sample. LOI was performed according to the ISO 4589-2:1996 standard using an HC-2-type instrument (China). Specimens with the dimension of 100 mm × 40 mm × 2 mm were used in all tests.

## 4. Conclusions

We found a higher amylose content leads to greater cohesion of starch gels as reflected by much higher tensile strength and fracture energy but does not influence *T*_S-G_. All the starch/Ca^2+^ gels displayed excellent adhesive properties on cardboard, and their adhesive force is all stronger than the cohesions within the corrugated paper. For wooden boards, the WCS/Ca^2+^, NCS/Ca^2+^, and G50/Ca^2+^ starch gels show excellent adhesive abilities, especially after enough storage, but not the G70/Ca^2+^ gel, which will peel off from the wood surface by itself. Moreover, the introduction of Ca^2+^ also imparts the starch gels with flame retardancy, with their limiting oxygen index (LOI) each being around 60. Therefore, these gels can bond paper or wood while providing a flame-retardant effect.

## Figures and Tables

**Table 1:** Molecular structure parameters of native starches.

	Amylopectin					Amylose		
Samples	Content (%)	*M*_w_ * (kDa)	Polydispersity (*M*_w_/*M*_n_)	*R*_z_ (nm)	Content (%)	*M*_w_ (kDa)	Polydispersity (*M*_w_/*M*_n_)	*R*_z_ (nm)
WCS	100%	77,068.05	1.553	130.51	0%	------	------	------
NCS	73%	61,194.34	2.417	117.95	27%	9068.29	1.011	126.56
G50	45%	55,962.61	1.452	107.28	55%	2060.63	1.714	92.919
G70	32%	35,458.19	1.363	105.72	68%	721.32	2.076	84.465

* *M*_w_ means weight-average molecular mass, *M*_n_ means number-average molecular mass, and *R*_z_ represents root mean square radius.

## Data Availability

Data may be shared under request.

## Supplementary Materials

The following supporting information can be downloaded at: https://www.mdpi.com/article/10.3390/molecules28114543/s1, Figure S1: Mass changes of the starch/Ca^2+^ gels stored at 84% relative humidity for 30 days; Figure S2: Steady rheological properties of the starch/Ca^2+^ colloidal solutions; Figure S3: Torn section photo of the bonded corrugated paper by the G50/Ca^2+^ gels; Figure S4: Torn section photos of the bonded wooden boards by the WCS/Ca^2+^ (a), NCS/Ca^2+^ (b), G50/Ca^2+^ (c), and G70/Ca^2+^ (d) gels after 1-day storage; Figure S5: Section photos of the bonded wooden boards by the WCS/Ca^2+^ (a), NCS/Ca^2+^ (b), G50/Ca^2+^ (c), and G70/Ca^2+^ (d) gels; Video Clip S1: flame-retardant application of the G50/Ca^2+^ gel in paper; Video Clip S2: flame-retardant application of the G50/Ca^2+^ gel in wood. References [39,40] are cited in the supplementary materials.

## Sample Availability

Samples of the compounds are available from the authors.
