# Supplementary material for: Adhesive and Flame-Retardant Properties of Starch/Ca2+ Gels with Different Amylose Contents
Source: Molecules. 2023 Jun 4;28(11):4543. doi: 10.3390/molecules28114543 (PMC10254862; doi:10.3390/molecules28114543)
Supplement: Supplementary file 1 [file molecules-28-04543-s001.zip › molecules-2427759-Supplementary.pdf]

§ Supporting Information §

Adhesive and flame-retardant properties of starch/Ca^{2+} gels with different amylose contents

Peng Liu[1,2], Jiandi Ling[1], Taoyan Mao[1], Feng Liu[3], Wenzhi Zhou[3], Guojie Zhang[*1], Fengwei Xie[*2]

[1] *School of Chemistry and Chemical Engineering, Guangzhou University, Guangzhou Higher Education Mega Center, Guangzhou 510006, China*

[2] *School of Engineering, Newcastle University, Newcastle upon Tyne NE1 7RU, UK*

[3] *Jiangsu Sanshu Biotechnology Co., Ltd., Nantong 226006, China*

[*] Corresponding authors. guojie.zhang@gzhu.edu.cn (G.Z.); david.xie@newcastle.ac.uk (F. X.)

Number of pages: 7

Number of figures: 5

1. Figures

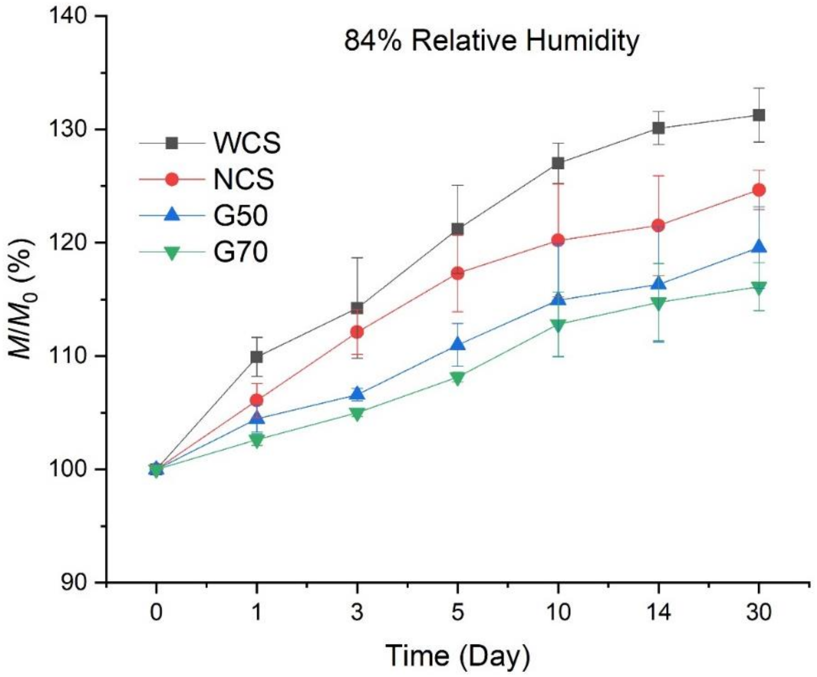

Figure. S1 Mass changes of the starch/Ca^{2+} gels stored at 84% relative humidity for 30 days.

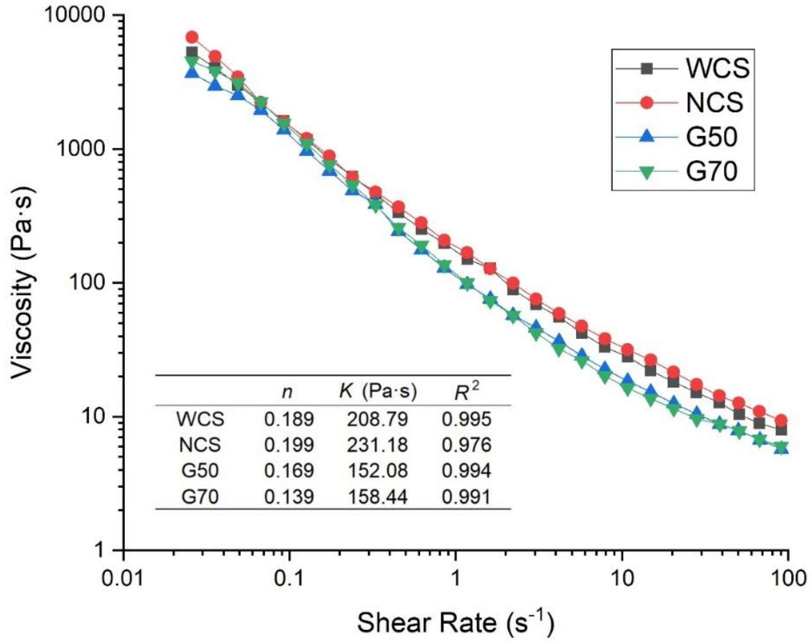

Figure. S2 Steady rheological properties of the starch/Ca^{2+} colloidal solutions.

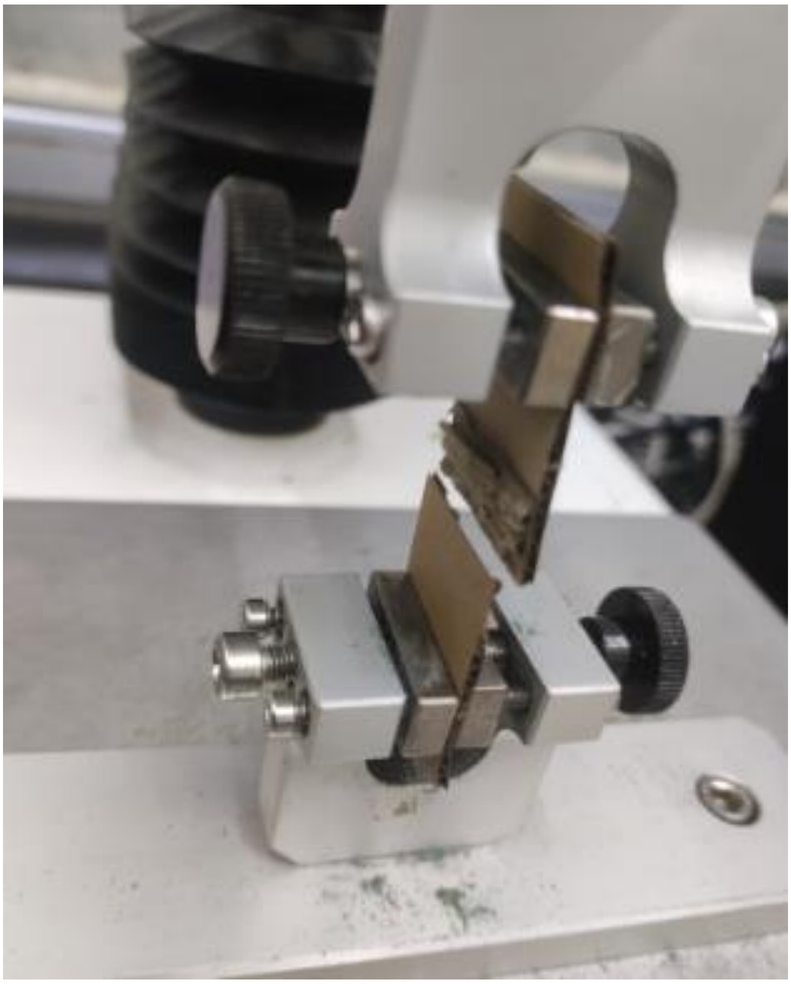

Figure. S3 Torn section photo of the bonded corrugated paper by the G50/Ca^{2+} gels.

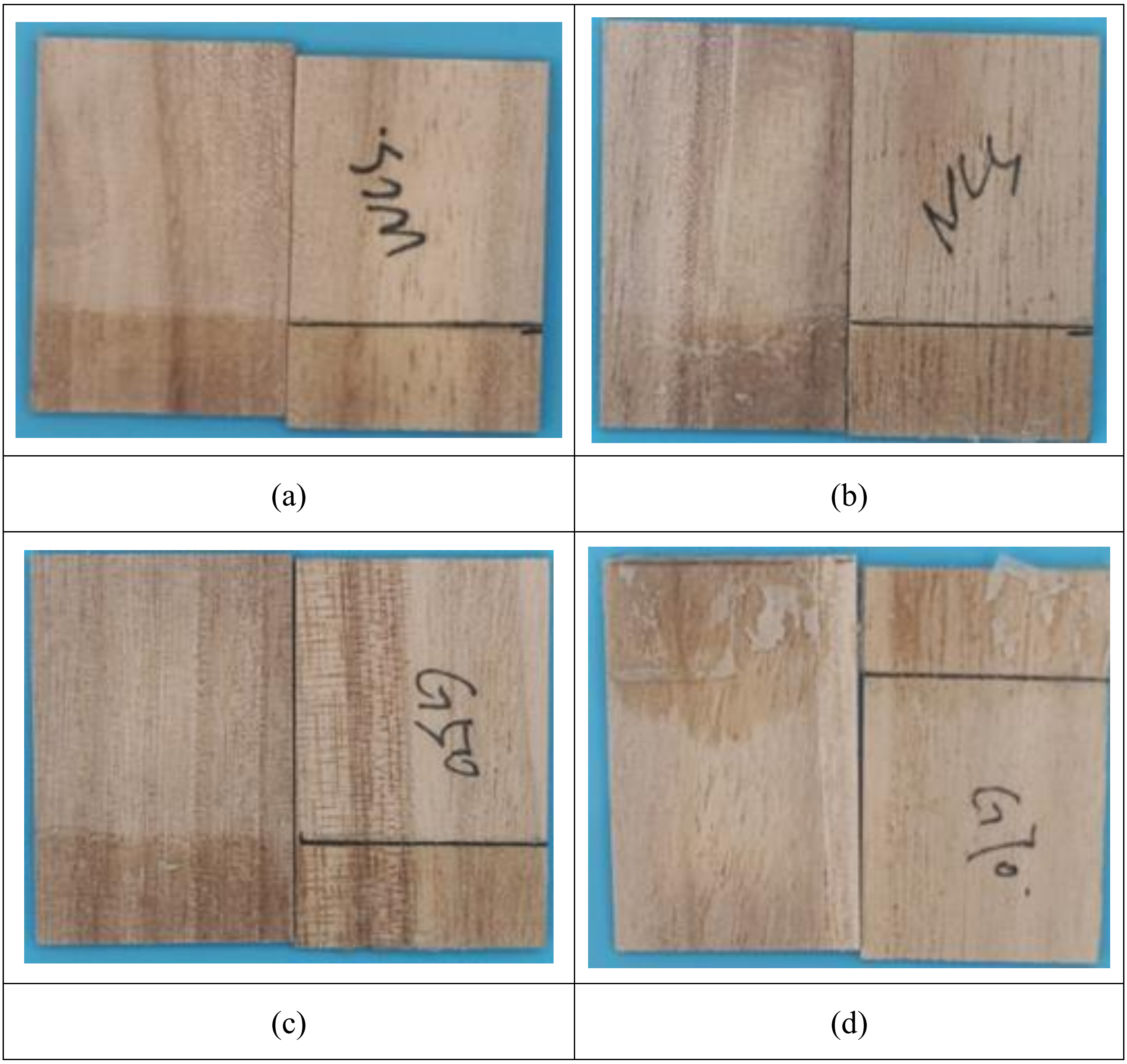

Figure. S4 Torn section photos of the bonded wooden boards by the WCS/Ca^{2+} (a), NCS/Ca^{2+} (b), G50/Ca^{2+} (c), and G70/Ca^{2+} (d) gels after 1-day storage.

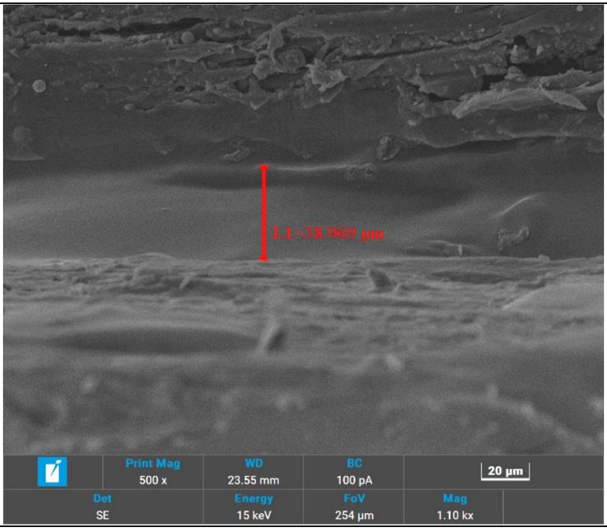

(a)

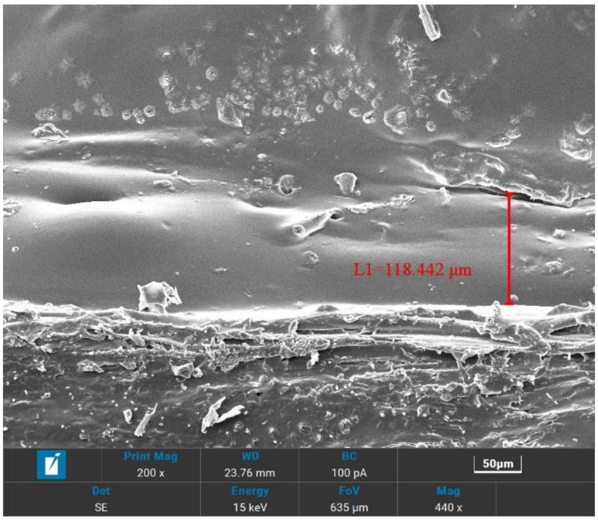

(b)

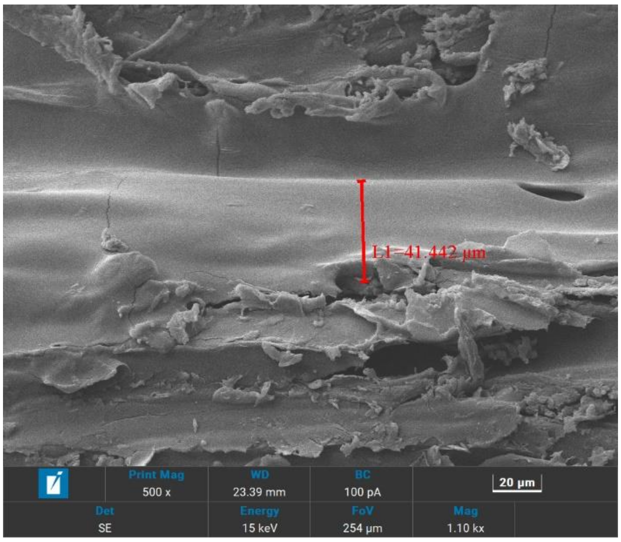

(c)

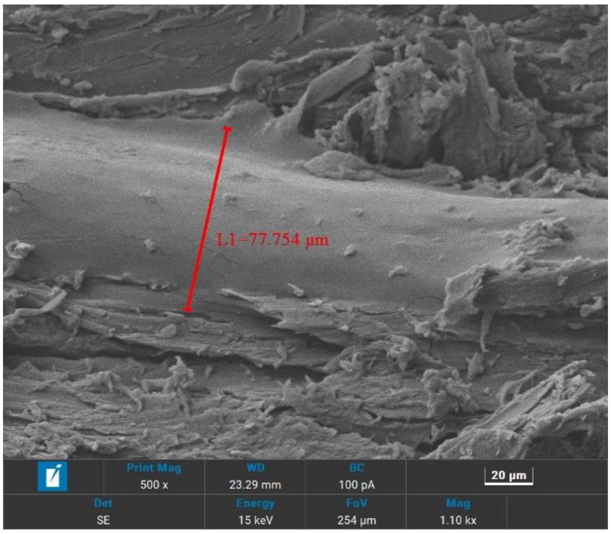

(d)

Figure. S5 Section photos of the bonded wooden boards by the WCS/Ca^{2+} (a), NCS/Ca^{2+} (b), G50/ Ca^{2+} (c), and G70/Ca^{2+} (d) gels.

2. Note to figures

Figure. S1 shows that at 84% RH, there is an increase in mass for all the four gels, which should be caused by a high concentration of Ca^{2+}. Moreover, at the same storage time, the mass of the

WCS/Ca^{2+} gel is the highest, followed by the NCS/Ca^{2+}, G50/Ca^{2+}, and G70/Ca^{2+} gels. This shows that the WCS/Ca^{2+} gel has the highest hydration ability, and those with higher amylose contents have a lower ability to absorb moisture from the air.

Figure. S2 shows that all the starch colloidal solutions exhibit shear-thinning behavior, and a linear relationship between viscosity and shear rate can be seen on the double logarithmic plot. The table in **Figure. S2** lists the parameters of the power-law model (**Equation. 1**), which were calculated from the curves in **Figure. S2**. Specifically, η is the viscosity (Pa·s) of the starch colloidal solution, $\dot{\gamma}$ is the shear rate (s^{-1}), K is the consistency (Pa·s), and n is the power-law index [1].

$$\eta = K\dot{\gamma}^{n-1} \quad \textbf{(1)}$$

Nearly all correlation coefficients (R^2) are higher than 0.97, showing a strong power-law dependence of viscosity on shear rate. Besides, the K values for WCS and NCS colloidal solutions are higher than those for G50/Ca^{2+} and G70/Ca^{2+} colloidal solutions, and thus in the static state, WCS/Ca^{2+} and NCS starch/Ca^{2+} colloidal solutions have a higher viscosity than G50 and G70 ones. Regarding this, at a low concentration, the viscosity of the colloidal solution is contributed more by the hydration between starch chains and water molecules, but less by the entanglement of starch chains. Since WCS and NCS have more amylopectin chains than G50 and G70 starches, and the hydration capability of amylopectin is much stronger than that of amylose, it is reasonable to observe higher K values for the WCS and NCS colloidal solutions.

For pseudo-plastic solutions, a smaller *n* value means a more significant effect of shear thinning. Linear macromolecular chains are more prone to show thinning [2]. The WCS and NCS starch colloidal solutions display higher *n* than the G50 and G70 colloidal solutions. This also proves a more ball-like conformation of amylopectin and a more line-like conformation of amylose.

3. References

[1] X. Chen, P. Liu, X. Shang, F. Xie, H. Jiang, J. Wang, Investigation of rheological properties and conformation of cassava starch in zinc chloride solution, Starch - Stärke 69(9-10) (2017) 1600384.

[2] W.L. Xie, L. Shao, Phosphorylation of Corn Starch in an Ionic Liquid, Starch-Starke 61(12) (2009) 702-708.
